# Supplementary material for: Companion Animals—An Overlooked and Misdiagnosed Reservoir of Carbapenem Resistance
Source: Antibiotics (Basel). 2022 Apr 17;11(4):533. doi: 10.3390/antibiotics11040533 (PMC9032395; doi:10.3390/antibiotics11040533)
Supplement: Supplementary file 1 [file antibiotics-11-00533-s001.zip › antibiotics-1678948-supplementary.pdf]

Companion Animals—An Overlooked and Misdiagnosed Reservoir of Carbapenem Resistance

Joana Moreira da Silva [1,2,†,‡], Juliana Menezes [1,2,†,‡], Cátia Marques [1,2,3,†] and Constança Ferreira Pomba [1,2,4,*,†]

1 Centre for Interdisciplinary Research in Animal Health (CIISA), Faculty of Veterinary Medicine, University of Lisbon, 1300-477 Lisbon, Portugal; jmsilva@fmv.ulisboa.pt (J.M.d.S.); julianamenezes@fmv.ulisboa.pt (J.M.); catia.marques@ulusofona.pt (C.M.)
2 Associate Laboratory for Animal and Veterinary Sciences (AL4AnimalS), 1300-477 Lisbon, Portugal
3 Faculty of Veterinary Medicine, Lusófona University, 1749-024 Lisbon, Portugal
4 Molecular Veterinary Diagnostic Laboratory-Genevet, 2790-140 Carnaxide, Portugal
* Correspondence: cpomba@fmv.ulisboa.pt
† Members of ENOVAT—European Network for Optimization of Veterinary Antimicrobial Treatment (COST ACTION CA18217).
‡ These authors contributed equally to this work.

Table S1. Representative MIC values for Imipenem, Meropenem and Ertapenem from companion animals Carbapenemase-producing isolates

Carbapenemase	Year	Country	Host	Source	Bacterial Species	Minimum Inhibitory Concentration (mg/L)			Ref.
						IMP	MEM	ETP	
KPC-2	2018	Brazil	Dog	Infection (UTI)	*Escherichia coli*	4	4	4	[9]
KPC-2	2021	Brazil	Dog	Infection (UTI)	*Klebsiella pneumoniae*	>32	>32	NA	[10]
KPC-4	2018	USA	Dog	Infection (UTI, SSTI)	*Enterobacter xiangfangensis*	≤1	2	NA	[11]
KPC-4	2018	USA	Dog	Infection (UTI, SSTI)	*Enterobacter xiangfangensis*	≤1	1	NA	[11]
NDM-1	2013	United States	Dogs and Cats	Infection (SSTI)	*Escherichia coli*	NA	1	NA	[24]
NDM-1	2013	United States	Dogs and Cats	Infection (UTI)	*Escherichia coli*	NA	0.5	NA	[24]
NDM-1	2013	United States	Dogs and Cats	Infection (UTI)	*Escherichia coli*	NA	4	NA	[24]
NDM-1	2013	United States	Dogs and Cats	Infection (UTI)	*Escherichia coli*	NA	16	NA	[24]
NDM-1	2017	China	Dog	Commensal	*Escherichia coli*	64	64	256	[25]
NDM-1	2017	China	Dog	Comensal	*Escherichia coli*	32	64	NA	[16]
NDM−1	2018	Italy	Dog	Comensal	*Acinetobacter radioresistens*	>32	>32	NA	[23]
NDM-5	2017	China	Dogs	Comensal	*Escherichia coli*	32	32	NA	[16]
NDM-5	2017	China	Dogs	Comensal	*Escherichia coli*	16	64	NA	[16]
NDM-5	2017	China	Dogs	Comensal	*Escherichia coli*	64	128	NA	[16]
NDM-5	2017	China	Dogs	Comensal	*Escherichia coli*	8	32	NA	[16]
NDM-5	2017	China	Dogs	Comensal	*Escherichia coli*	16	32	NA	[16]
NDM-5	2017	China	Dogs	Comensal	*Escherichia coli*	8	16	NA	[16]

NDM-5	2018	United States	Dog	Infection (URTI)	*Escherichia coli*	4	NA	NA	[20]
NDM-5	2019	South Korea	Dog	Commensal	*Escherichia coli*	4	4	16	[21]
NDM-5	2019	South Korea	Cats	Commensal	*Escherichia coli*	16	32	>32	[21]
NDM-5	2019	United Kingdom	Dog	Infection (SSTI)	*Escherichia coli*	4	4	NA	[19]
NDM-5	2021	Italy	Dog	Infection (UTI)	*Escherichia coli*	>16	>16	>2	[15]
NDM-9	2017	China	Dogs	Comensal	*Escherichia coli*	8	64	NA	[16]
OXA-48	2017	France	Dog	Commensal	*Escherichia coli*	1.5	0.75	0.75	[30]
OXA-181	2018	Switzerland	Dogs	Commensal	*Escherichia coli*	1	0.5	>2	[26]
OXA-181	2018	Switzerland	Dogs and Cats	Commensal	*Escherichia coli*	0.5	0.5	>2	[26]
OXA-181	2018	Switzerland	Dogs and Cats	Commensal	*Escherichia coli*	0.5	0.5	2	[26]
OXA-181	2018	Switzerland	Dogs and Cats	Commensal	*Escherichia coli*	0.5	0.25	2	[26]
OXA-181	2018	Switzerland	Dogs	Commensal	*Escherichia coli*	0.5	1	>2	[26]
OXA-181	2018	Switzerland	Cat	Commensal	*Escherichia coli*	0.25	0.25	2	[26]
OXA-181	2020	Portugal	Dog	Commensal	*Escherichia coli*	≤1	≤1	1	[27]
OXA-23	2014	Portugal	Cat	Infection (UTI)	*Acinetobacter baumannii*	>8	>8	NA	[33]
OXA-23	2017	Germany	Dogs and Cats	Infection (UTI, SSTI, URTI, CRBSI, suppurate inflammation)	*Acinetobacter baumannii*	>16	NA	NA	[34]
OXA−23	2018	Italy	Dogs and Cats	Comensal	*Acinetobacter baumanni*	>32	>32	NA	[23]
OXA−23	2018	Italy	Dogs and Cats	Comensal	*Acinetobacter baumanni*	13	>32	NA	[23]
OXA-23	2014	Portugal	Cat	Infection (UTI)	*Acinetobacter baumannii*	>8	>8	NA	[33]
VIM-1	2016	Spain	Dog	Commensal	*Klebsiella pneumoniae*	4	4	≤2	[14]

ETP, Ertapenem; IMP, Imipenem; MEM, Meropenem; NA, not applicable.
